# Pandemic Spread—an Empirical Analysis

**DOI:** 10.5041/RMMJ.10410

**Published:** 2020-07-31

**Authors:** Zvi Ziegler

**Affiliations:** Professor Emeritus, Department of Mathematics, Technion–Israel Institute of Technology, Haifa, Israel

**Keywords:** Infection rate, pandemic, public awareness, restrictions

## Abstract

The coronavirus disease-2019 (COVID-19) epidemic started in late 2019, and was upgraded to a pandemic on March 11, 2020 by the World Health Organization (WHO). Well established epidemiological models have been used over the last few months in an attempt to predict how the virus would spread. The predictions were frightening, and the resulting panic caused many governments to impose lockdowns or other severe restrictions, with lasting effects. This short paper discusses another way of looking at the spread of COVID-19, by focusing on the daily rate of infection, defined as the daily rate of increase in the number of infected persons. It is shown that the daily rate is monotonically decreasing, after a short initial period, in all countries, and that the pattern is similar in all countries. This appears to be a universal phenomenon. Based on these calculations, the April 1, 2020 data for Western Europe were sufficient to predict the beginning of the end of COVID-19 in that region before the end of that month.

## INTRODUCTION

At the time of writing, in May 2020, the coronavirus disease-2019 (COVID-19) pandemic has been with us for more than 5 months, and the available data allow analysis of the rate of spread. Analytical tools would be useful for decision makers, both now and for the future, if there is a second wave.

The most serious problem in this context is the reliability and the meaning of the available data, both as to the number of infected persons and to the number of deaths due to COVID-19. Moreover, the percentage of people who have been tested, the methodology for choosing who is tested, and how, all vary between countries.

We might think that the number of deaths is iron-clad and totally reliable for comparative evaluation, but even the definitions for attributing deaths to COVID-19 are not identical in all countries.^1^ For example, in some countries, the death of a person who has not been previously identified as being infected will not be attributed to the virus. In other countries, if COVID-19 is detected at autopsy the death is counted as due to the virus, whether or not the virus actually caused the death or was simply present. Moreover, the definitions for a COVID-19-related death changed in the same country, often for political reasons (France, UK, and the USA are prominent examples).^2^,^3^

In view of these ambiguities all international comparisons are to be viewed with caution. I have chosen a different route, by considering the trends over time in each country separately. The way each country operates over time is generally consistent, and therefore the analysis of trends over time is reasonable, unless there are internal changes of definitions or methodologies. In such a case, the trends before and after such changes should be viewed separately. An interesting example occurred in Ireland in the week of April 8–15, 2020, where there seemed to be a “deviation” from the trend. On further investigation, it was discovered that large batches of COVID-19 tests had been sent to Germany for testing, resulting in an abrupt change in the number of persons who tested positive that week.^4^ Caution is the name of the game; nevertheless, in my opinion, the in-country trends are the most reliable way to assess the spread of this coronavirus.

Herein I discuss an innovative calculation for looking at the spread of COVID-19, by focusing on the daily rate of infection, defined as the daily rate of increase in the number of infected persons. This calculation is shown to have been sufficient, on the week ending April 1, to predict the beginning of the end of COVID-19 in Western Europe before the end of April, 2020.

## METHODOLOGY FOR THE EMPIRICAL STUDY OF INFECTION RATES

To better understand the COVID-19 pandemic and the actual rate of infection, I began to look at raw data as it became available, using worldometer.com.^5^ Initially, I compared their data to that of the Johns Hopkins tracker^6^ as well—with the same results. I therefore focused on using worldometer.com as the most appropriate data source for my study, since the fixed daily cut-off time of GMT +0 ensures that data can be easily compared. This provided consistency for dealing with comparisons over time.

My approach is different from standard analyses of epidemics, was based on easily obtained data, and is, to the best of my knowledge, my own innovative contribution. Table 1 includes the average daily infection rate for each week since March 4, 2020 for most Western European countries where the outbreak occurred earlier. There were two reasons behind the decision to initially examine the data for Western European countries starting from March 2020: firstly, data from Europe for February were erratic, and an apparatus for testing and reporting was only just getting started. Secondly, COVID-19 was already widespread there when this study began on March 4, 2020, and although diagnostic definitions were not identical, the reporting of data from these nations was generally trustworthy. On March 11, 2020, the disease was designated by the World Health Organization (WHO) as a pandemic. Public awareness of this dangerous disease was awakened in other parts of the world, and this led, at a later stage, to an extension of my study to include several other countries.

**Table 1:** Calculated Daily Infection Rate for Weeks of February 26 through April 1, 2020. Based on actual data from worldometers.com^5^ and calculation of *I* (*n*) (see text). My prediction, made and widely disseminated at the beginning of April (see text), was that the rate of increase would be below 1.05 for most of Western Europe before the end of April.

Country	Feb. 26–March 4	March 4–11	March 11–18	March 18–25	March 25–April 1
Austria	--	1.36	1.31	1.19	1.09
Belgium	--	1.45	1.25	1.19	1.16
Denmark	--	1.67	1.11	1.07	1.09
Finland	--	1.38	1.28	1.14	1.07
France	--	1.34	1.22	1.16	1.12
Germany	--	1.33	1.30	1.17	1.11
Greece	--	1.41	1.23	1.10	1.08
Iceland	--	1.20	1.18	1.13	1.06
Ireland	--	1.32	1.36	1.23	1.12
Israel	--	1.30	1.24	1.27	1.14
Italy	1.31	1.22	1.16	1.11	1.06
Luxembourg	--	1.32	1.61	1.31	1.08
Netherlands	--	1.44	1.22	1.18	1.11
Norway	--	1.40	1.14	1.10	1.06
Portugal	--	1.39	1.40	1.25	1.16
Spain	--	1.39	1.30	1.17	1.11
Sweden	--	1.38	1.15	1.10	1.10
Switzerland	--	1.32	1.25	1.20	1.07
United Kingdom	--	1.27	1.28	1.20	1.17
United States	--	1.35	1.32	1.33	1.18

--, available data inadequate for meaningful calculations.

The numbers considered are the numbers of infected people, i.e. the numbers of people who tested positive for the virus. Tables 1 and 2 describe the average daily infection rate in each week. Table 1 describes the findings until April 1 that formed the basis for predictions, explained below. Table 2 describes the findings until May 13, for an expanded list of countries and states. To create these tables, I needed, firstly, to develop a definition of the daily rate of increase. Since usually there are some fluctuations during any given week, I chose to calculate and present in the tables the average rate during each week.

**Table 2:** Average Daily Infection Rate for Weeks of March 4 through May 13, 2020. Based on actual data from worldometers.com^5^ and calculation of *I* (*n*) (see text). Values below 1.01 are presented to 3 digits, to show how far the calculation is below 1.

Country	Data from Table 1, Used to Predict a Rate of Increase Below 1.05 by the end of April, 2020					Actual Calculated Data, Demonstrating Correctness of Prediction Based on Table 1				
	March 4–11	March 11–18	March 18–25	March 25–April 1	April 1–8	April 8–15	April 15–22	April 22–29	April 29–May 6	May 6–13
Australia	--	1.25	1.24	1.10	1.03	1.01	1.004	1.002	1.003	1.002
Austria	1.36	1.31	1.19	1.09	1.03	1.01	1.01	1.005	1.003	1.003
Belarus	--	--	--	--	1.31	1.20	1.10	1.09	1.06	1.04
Belgium	1.45	1.25	1.19	1.16	1.08	1.05	1.03	1.02	1.009	1.009
Canada	--	1.31	1.25	1.16	1.10	1.06	1.05	1.04	1.03	1.02
Denmark	1.67	1.11	1.07	1.09	1.08	1.03	1.02	1.02	1.01	1.01
Finland	1.38	1.28	1.14	1.07	1.08	1.04	1.04	1.02	1.02	1.03
France	1.34	1.22	1.16	1.12	1.10	1.04	1.02	1.01	1.007	1.01
Germany	1.33	1.30	1.17	1.11	1.05	1.03	1.02	1.01	1.006	1.005
Greece	1.41	1.23	1.10	1.08	1.04	1.02	1.01	1.01	1.005	1.005
Iceland	1.20	1.18	1.13	1.06	1.04	1.01	1.005	1.001	1.00	1.00
Ireland	1.32	1.36	1.23	1.12	1.08	1.11	1.04	1.03	1.01	1.007
Israel	1.30	1.24	1.27	1.14	1.06	1.04	1.02	1.01	1.004	1.002
Italy	1.22	1.16	1.11	1.06	1.03	1.02	1.02	1.01	1.007	1.005
Luxembourg	1.32	1.61	1.31	1.08	1.04	1.02	1.01	1.01	1.003	1.002
Mexico	--	--	1.23	1.17	1.13	1.10	1.08	1.08	1.07	1.05
Netherlands	1.44	1.22	1.18	1.11	1.06	1.05	1.03	1.02	1.009	1.006
Norway	1.40	1.14	1.10	1.06	1.03	1.02	1.01	1.01	1.005	1.003
Poland	--	1.37	1.20	1.14	1.11	1.06	1.04	1.03	1.02	1.02
Portugal	1.39	1.40	1.25	1.16	1.07	1.05	1.03	1.02	1.01	1.01
Romania	--	1.28	1.20	1.15	1.10	1.06	1.04	1.03	1.02	1.02
Russia	--	--	1.23	1.23	1.18	1.16	1.13	1.08	1.08	1.06
Spain	1.39	1.30	1.17	1.11	1.05	1.03	1.02	1.02	1.01	1.01
Sweden	1.38	1.15	1.10	1.10	1.08	1.05	1.04	1.03	1.02	1.02
Switzerland	1.32	1.25	1.20	1.07	1.04	1.02	1.01	1.01	1.003	1.002
UK	1.27	1.28	1.20	1.17	1.11	1.07	1.04	1.03	1.03	1.02
USA	1.35	1.32	1.33	1.18	1.11	1.06	1.04	1.03	1.02	1.02
CA	--	--	1.20	1.18	1.10	1.05	1.05	1.04	1.03	1.03
FL	--	--	1.29	1.22	1.11	1.05	1.03	1.02	1.02	1.02
GA	--	--	1.32	1.19	1.12	1.06	1.05	1.03	1.03	1.02
LA	--	--	1.30	1.20	1.15	1.04	1.02	1.01	1.01	1.01
NJ	--	--	1.46	1.3	1.15	1.07	1.05	1.03	1.04	1.01
NY	--	--	1.44	1.15	1.09	1.05	1.03	1.02	1.01	1.01
PA	--	--	1.33	1.27	1.16	1.07	1.05	1.03	1.03	1.02
TX	--	--	1.29	1.20	1.14	1.07	1.04	1.04	1.04	1.03
WA	--	--	1.12	1.12	1.07	1.02	1.02	1.02	1.02	1.01

--, available data inadequate for meaningful calculations; CA, California; FL, Florida; GA, Georgia; LA, Louisiana; NJ, New Jersey; NY, New York; PA, Pennsylvania; TX, Texas; UK, United Kingdom; USA, United States of America; WA, Washington.

The daily rate of infection is calculated as follows:

(Eq. 1) $$\frac{A(n+1)}{A(n)}=I(n)$$

where: *A*(*n*) is the number of infected persons on the *n*-th day; *A*(*n* + 1) is the number of infected persons on day *n* + 1; and *I* (*n*) the rate of increase at the *n*-th day.

I was interested in obtaining *I* (*n*) and determining its behavior over time. If *I* (*n*) = *I* (1), for all *n*, i.e. there is no change over time, then the number of infected persons would be expected to increase in a geometric progression, compounded daily by a factor of *I* (1). For example, if *I* (*n*) = 1.26 (approximately), for all *n*, the number of infected people would be expected to double every three days.

The initial rates, before the public and the authorities were aware of the existence of a lethal disease and took precautions, were often close to, or even higher, than this number and led to fear of an uncontrollable exponential growth. However, as the calculations in Tables 1 and 2 demonstrate, *I* (*n*) decreases monotonically within a few weeks, and converges to 1. When *I* (*n*) is close to 1, the growth rate is close to linear, indicating that the disease is in decay, and the country can begin to relax any restrictions in force.

As explained above, since there are daily fluctuations in the infection rates, it was decided to consider the average daily increase rates for each week. An entry in Tables 1 and 2 was computed as follows:

(Eq. 2) $$\sqrt[{7}]{\frac{Total\;infected\;persons\;at\;end\;of\;week}{Total\;infected\;persons\;at\;beginning\;of\;week}}=Average\;Daily\;Increase\;for\;that\;week$$

where $\sqrt[{7}]{\;}$ is the seventh root of the weekly increase (one week has seven days).

The rationale behind using the above geometric progression to explore the spread of COVID-19 seemed natural to me, since we are all interested in how fast a given population is being infected. Since each day presents with new numbers, infection must be viewed from the beginning of each day by considering the total number of infected people. Theoretically, this should provide the rate at which that number will increase.

## RESULTS

Looking at my calculations based on the actual data of March 25, a clear pattern was discovered: a monotonic decrease of the average daily rate of increase in the number of infected persons. On that basis, the April 1 data for Western European countries, Israel (where I live), and the USA were examined, and were summarized in Table 1. This table was sent, with an analysis, to many colleagues, friends, and people close to the decision makers in Israel, on April 5, 2020. I included a *prediction*, that the rate of increase of the total number of COVID-19 infections would decrease, before the end of April, to below 1.05 (a daily rate of increase in the total number of infected persons of 1.05 is equivalent to a doubling of the number of infected persons after two weeks) in most of Western Europe, which would enable most of the countries to start relaxing restrictions. From Table 2, it is clear that this prediction was absolutely correct.

I subsequently added more countries outside of Western Europe to the analysis: Poland, Romania, Belarus (Eastern Europe), Russia, Canada, Australia, and Mexico. Since the USA is such a large and varied country, with each state having its own government, and interstate travel being unrestricted, it was interesting to include separate states. For the purposes of this study, I included nine states from different regions. Table 2 demonstrates that the patterns discovered for Western Europe were also valid in all 27 countries studied, and in the nine American states. There was a time lag in the downward convergence in some countries, depending on the time the public and the authorities decided to take the pandemic seriously. Sometimes this happened when the first few fatalities occurred, but it is difficult to determine the exact point in time when heightened public awareness began.

## DISCUSSION

Most assessments of epidemics use the *R_0_* statistic, i.e. the expected number of secondary cases produced by a single (typical) infection.^7^ However, the actual calculation of *R_0_* is quite difficult, since estimates depend on assumptions that are often quite hard to validate.^8^ I have proposed an alternative and easier methodology for calculating the spread of a pandemic.

The spread of an infection depends, among other things, on anthropological-sociological aspects.^9^ The infection process, at the initial and most important stages, is culture-dependent, according to some theories, and was recently discussed as it relates to COVID-19.^10^ An interesting example is the behavior of soccer-fans in and after the game between Atalanta-Bergamo and Valencia, on February 19, 2020, which was a very important factor in the initial spread in Italy and Spain.^11^

The way in which a community functions, the population density, the nature of social interaction, etc., are important. For example, there are significant differences between Scandinavian and Mediterranean countries such as Italy and Spain in this respect. A country with one major densely populated metropolitan area is different from a country with multiple foci.

After the initial stages, when the infection is getting attention, and the public becomes aware of the need to exercise caution due to a dangerous disease, the rate of infection begins to decrease. The reasons for the decrease are not fully understood, and deserve to be studied in depth. One possible explanation of the decrease states that infection occurs in two ways. Firstly, at the initial and crucial phase, it evolves within the social circle of the infected person and then decreases as infected people meet other infected people within their social circles.^12^ Secondary to this is the random community spread of infection within the public domain, as occurred in Barcelona, Spain.^11^ Anthropologists, sociologists, and behavioral scientists will undoubtedly collaborate in deciphering the relevant modes of behavior. In parallel, epidemiologists and virologists still have a lot more to research and learn about COVID-19, how it is transmitted, and its potency over time. Clearly, the spread of COVID-19 is highly dependent on both the virus itself and on human behavior.

**Figure 1:**
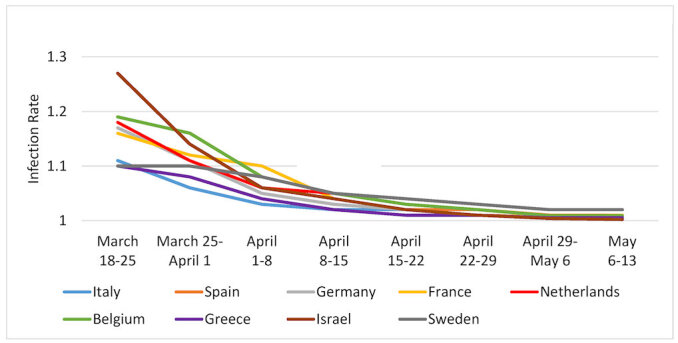
Rates of Pandemic Spread for Western Europe and Israel: March 18–May 13, 2020.

**Figure 2:**
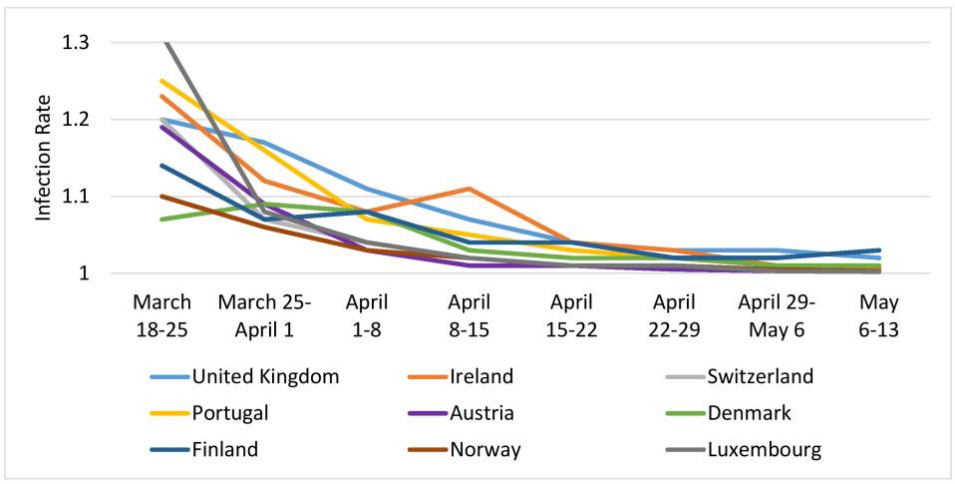
Rates of Pandemic Spread for Additional Western European Countries: March 18–May 13, 2020.

**Figure 3:**
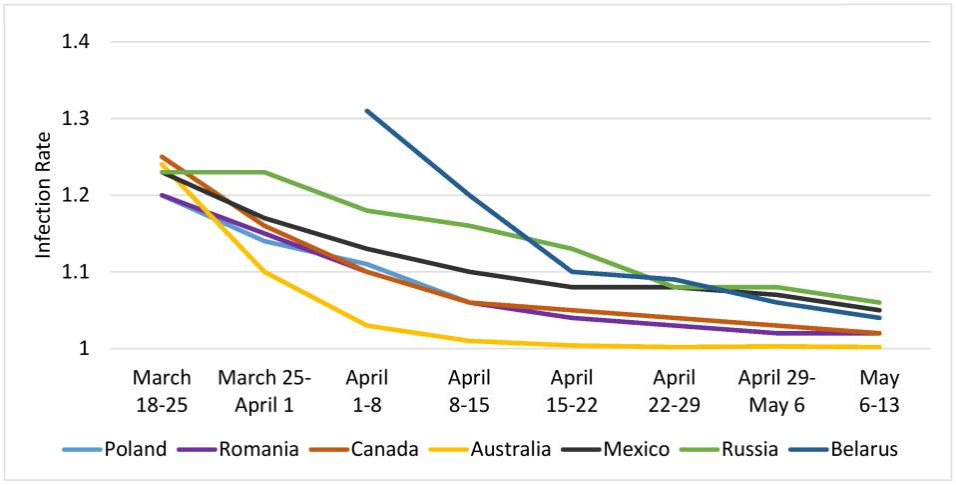
Rates of Pandemic Spread Outside of Western Europe: March 18–May 13, 2020 The line representing Poland is hidden by the data for Romania, reflecting similar data.

**Figure 4:**
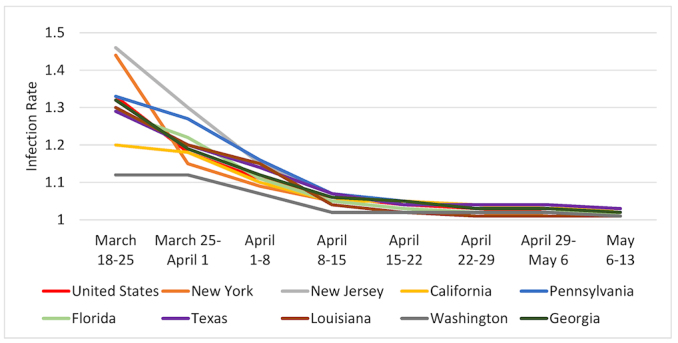
Rates of Pandemic Spread for the USA and Selected States: March 18–May 13, 2020.

Figures 1–4 are based on Table 2 and demonstrate the steady decrease of the rate of infection after the initial infection stage in all the examined countries (and nine states). A rate of infection close to 1 means that the exponential growth at that point in time was very close to the linear growth. Hence, if the rate of infection were, for example, 1.02, it would take more than a month to double the number of infected persons, making treatment more easily manageable. Moreover, at that rate of infection, the number of people recovering would exceed those who became infected during that period. In fact, there were several countries in Europe where this indeed started to happen in late April.

The daily rate of increase, *I* (*n*), is an important indication of the spread of COVID-19. This calculation is easily obtained, and an analysis of its behavior may serve as a predictor for the spread of the contagion, and help to improve healthcare system preparedness. It is also possible to draw conclusions regarding the need and timing for severe restrictions. Most countries listed in Tables 1 and 2 imposed restrictions on their populations, ranging from minor to highly restrictive. A comparison between Belgium, which imposed very severe restrictions very early, and Sweden, which relied more upon the residents’ understanding and compliance, reveals that the severity of restrictions was not the crucial factor. Belarus imposed no restrictions, but the infection rate followed the same pattern. Based on these data, my conclusions are that it is crucial to raise public awareness as early as possible. Moreover, while some restrictions may be required, such as restricting entrance into a country, prohibiting mass gatherings, and restricting activities where proper behavior is almost impossible, the extent of the restrictions should be limited and further relaxed once the country enters a “comfort zone,” with the infection rate falling below 1.05. Relaxing restrictions should be accompanied by a campaign exhorting people to behave responsibly in the public and private spheres, in order to prevent complacency.

## CONCLUSION

The geometric rate of increase model, proposed herein, is much easier to evaluate than the statistic *R_o_*, which is widely used. It circumvents the difficulties in calculating *R_0_*, and is potentially a useful tool for analyzing the spread of the COVID-19 pandemic as well as other pandemics. Estimations should be calculated on future data of the current pandemic, and a more comprehensive approach to assessing the spread of a pandemic, utilizing both *R_o_* and the geometric rate of increase model, may be explored.

## Abbreviations

COVID-19: coronavirus disease-2019
USA: United States of America
WHO: World Health Organization.

## References

[1] Morris C, Reuben A (2020). Coronavirus: why are international comparisons difficult?. BBC News.

[2] The Economist (2020). Tracking covid-19 excess deaths across countries.

[3] Brown E, Reinhard B, Thebault R (2020). Which deaths count toward the covid-19 toll? It depends on the state. The Washington Post.

[4] Murray S (2020). Irish Air Corps makes first delivery of Covid-19 tests to German lab. The journal.ie news.

[5] Worldometers website. COVID-19 coronavirus pandemic.

[6] Johns Hopkins University & Medicine website.

[7] Ridenhour B, Kowalik JM, Shay DK (2014). Unraveling R_0_: considerations for public health applications. Am J Public Health.

[8] Viceconte G, Petrosillo N (2020). COVID 19 R_0_: magic number or conundrum?. Infect Dis Rep.

[9] Manderson L (1998). Applying medical anthropology in the control of infectious disease. Trop Med Int Health.

[10] Van Bavel JJ, Baicker K, Boggio PS (2020). Using social and behavioural science to support COVID-19 pandemic response. Nat Hum Behav.

[11] Associated Press (2020). A ‘biological bomb’: Atalanta vs. Valencia in Milan linked to accelerating coronavirus outbreak. Sports Illustrated.

[12] Raffaetà R (2020). An anthropological perspective on viruses. Aspenia online | International Analysis and Commentary website.

